# Lentiviral Nef suppresses iron uptake in a strain specific manner through inhibition of Transferrin endocytosis

**DOI:** 10.1186/1742-4690-11-1

**Published:** 2014-01-02

**Authors:** Herwig Koppensteiner, Kristin Höhne, Marcos Vinicius Gondim, Francois-Xavier Gobert, Miriam Widder, Swantje Gundlach, Anke Heigele, Frank Kirchhoff, Michael Winkler, Philippe Benaroch, Michael Schindler

**Affiliations:** 1 Institute of Virology, Helmholtz Zentrum Munich, German Research Center for Environmental Health, Neuherberg, Germany; 2 Heinrich Pette Institute, Leibniz Institute for Experimental Virology, Hamburg, Germany; 3 Institut Curie, INSERM U932, Paris, France; 4 Ulm University Medical Center, Institute of Molecular Virology, Ulm, Germany; 5 German Primate Center, Göttingen, Germany; 6 Current address: Martin Luther University Halle-Wittenberg, Internal Medicine IV, Halle, Germany

**Keywords:** HIV, SIV, Nef, Iron homeostasis, AIDS, Lentiviral replication, Macrophages

## Abstract

**Background:**

Increased cellular iron levels are associated with high mortality in HIV-1 infection. Moreover iron is an important cofactor for viral replication, raising the question whether highly divergent lentiviruses actively modulate iron homeostasis. Here, we evaluated the effect on cellular iron uptake upon expression of the accessory protein Nef from different lentiviral strains.

**Results:**

Surface Transferrin receptor (TfR) levels are unaffected by Nef proteins of HIV-1 and its simian precursors but elevated in cells expressing Nefs from most other primate lentiviruses due to reduced TfR internalization. The SIV Nef-mediated reduction of TfR endocytosis is dependent on an N-terminal AP2 binding motif that is not required for downmodulation of CD4, CD28, CD3 or MHCI. Importantly, SIV Nef-induced inhibition of TfR endocytosis leads to the reduction of Transferrin uptake and intracellular iron concentration and is accompanied by attenuated lentiviral replication in macrophages.

**Conclusion:**

Inhibition of Transferrin and thereby iron uptake by SIV Nef might limit viral replication in myeloid cells. Furthermore, this new SIV Nef function could represent a virus-host adaptation that evolved in natural SIV-infected monkeys.

## Background

Iron is an essential element in the human body and involved in cellular proliferation and immune response [1]. Since free iron generates harmful reactive oxygen species iron homeostasis is tightly regulated. Dietary iron is resorbed by the Divalent Metal Transporter into enterocytes. Loaded on Transferrin (Tf) iron enters the blood stream and is taken up by target cells via the Transferrin Receptor I (TfR). Internalization of TfR can be antagonized by the hemochromatis protein (HfE). Thus, cells can regulate iron uptake by TfR expression or by the rate of TfR internalization through HfE [1,2]. Iron export from cells is also controlled by two antagonizing mechanisms. Ferroportin loads cellular iron on plasma Tf and this process is suppressed by the hormone hepcidine [3]. All these mechanisms have the consequence that only trace amounts of iron, the so called labile iron pool (LIP) [4], is redox-active. Long term storage of iron is within Ferritin, mainly in hepatocytes [1,5].

Dysregulation of iron homeostasis is a hallmark of many diseases including AIDS [1-3]. Elevated cellular iron loads are associated with high HIV-1 titers and faster progression to AIDS and iron is important in various steps of HIV-1 propagation [2,6-8]. Hence, viruses have evolved mechanisms to increase cellular iron [2]. The HIV-1 Nef protein was proposed to downregulate HfE, leading to enhanced uptake of iron loaded Tf [9].

HIV-1 Nef is considered as a pathogenicity factor contributing to AIDS progression [10]. It enhances viral replication and infectivity and mediates immune evasion by multiple functions, including downmodulation of CD4 and MHCI. Nef is highly variable and not only present in HIV-1, but also in simian immunodeficiency viruses (SIV) and HIV-2 [10]. HIV-1 causes AIDS in humans and it was demonstrated that SIVcpz leads to immunodeficiency in chimpanzees [11]. HIV-2 is usually less pathogenic than HIV-1 but causes AIDS post an extended chronical phase [12]. Within the natural simian hosts, SIVs do not cause disease due to a well balanced virus-host coevolution. Hallmarks of non-pathogenic SIV infections are high viral loads in the absence of chronic and generalized immune activation [13]. Notably, Nef proteins derived from non-pathogenic infections prevent activation of infected T cells by removal of the CD3 molecule from the cell surface [14]. Thus, SIV Nef could be considered as a “persistence” factor in contrast to its HIV-1 counterpart.

We hypothesized that lentiviral Nefs may also differ in their ability to manipulate cellular iron uptake. Interestingly, we found that Nef-mediated degradation of HfE is not a conserved feature of HIV-1 and other lentiviral Nefs. In contrast, we demonstrate inhibition of TfR internalization by most SIV Nef proteins including the lentiviruses which are non-pathogenic in their natural simian hosts. This phenotype results in reduced cellular iron levels and attenuated lentiviral replication in macrophages. Negative regulation of cellular iron stores by Nef in infected cells might be an additional strategy to achieve non-harmful and persistent virus-host coexistence.

## Results

### SIV Nef increases the cell surface expression of TfR

**Figure 1:**
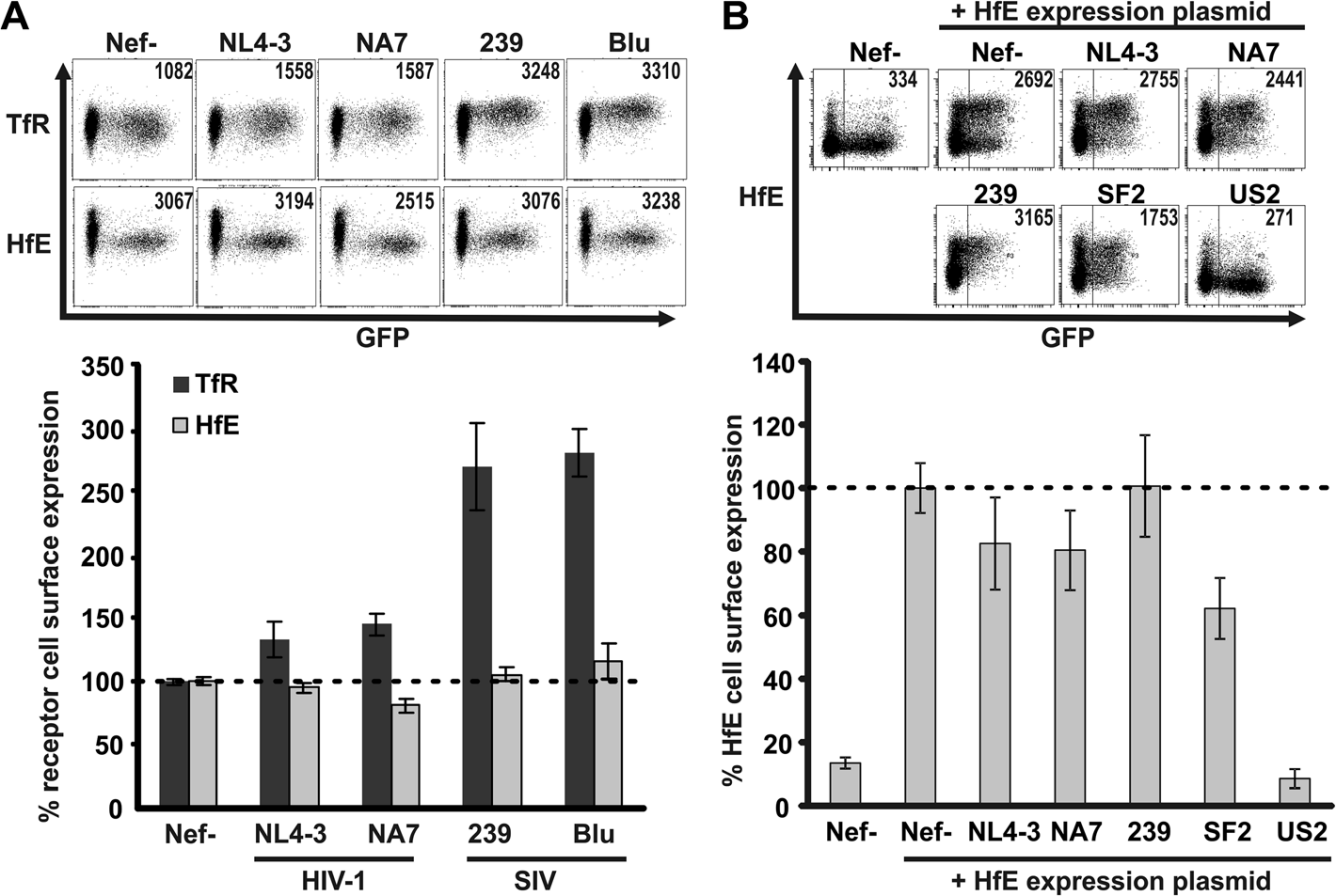
**Effects of Nef expression on cell surface levels of TfR and HfE. (A)** THP-1 cells were infected with HIV-1 coexpressing different Nef proteins and GFP via an IRES. 48 hours post infection cells were surface stained with antibodies against TfR or HfE and analysed by flow cytometry. Depicted are mean values and standard deviation (SD) from three independent experiments. **(B)** 293 T cells were transfected with pCG-IRES-GFP plasmids expressing the indicated Nef and cotransfected with an HfE expression plasmid. 36 hours later cells were harvested and cell surface levels of HfE were assessed by antibody staining and flow cytometry. Mean values and SD are derived from three independent experiments.

We assessed if modulation of receptors involved in cellular iron uptake is a conserved feature of different lentiviral Nef variants. Macrophages regulate iron turnover *in vivo*[1,15]. Therefore, we infected the myeloid cell line THP-1 with HIV-1 coexpressing Nef and eGFP (HIV-NIG) [14] via an internal ribosomal entry site (IRES) and measured cell surface expression of TfR and HfE by flow cytometry (Figure 1). Both remained largely unchanged upon expression of HIV-1 Nef (Figure 1A). In contrast, Nef from SIVmac239 or the distantly related SIVblu caused up to threefold increase in TfR levels without modifying HfE surface levels (Figure 1A).

Drakesmith and colleagues reported degradation of HfE by HIV-1 SF2 Nef in HeLa cells transfected to express HfE [9]. Although HIV-1 NA7, NL4-3 and SF2 Nef are highly similar, we considered the possibility that subtle alterations in Nef might be sufficient to allow HfE degradation. Consistent with the results from HIV-1 infected THP-1 cells (Figure 1A), NL4-3, NA7 or 239 Nef had no or only marginal effects on cell surface HfE upon cotransfection in 293 T cells with HfE cDNA (Figure 1B). 293 T cells transfected with the HfE plasmid showed a strong increase in cell surface staining, demonstrating the specificity of the HfE antibody and SF2 Nef downmodulated approximately 40% of cell surface HfE under these experimental conditions (Figure 1B). In agreement with its reported capacity to induce HfE degradation [16], expression of the HCMV US2 protein led to a drastic reduction of HfE surface levels (>90%) (Figure 1B). Thus, modulation of HfE is not a conserved feature of lentiviral Nefs. Conversely, SIV Nef enhances cell surface levels of TfR.

### Most SIV Nef proteins increase cell surface TfR

**Figure 2:**
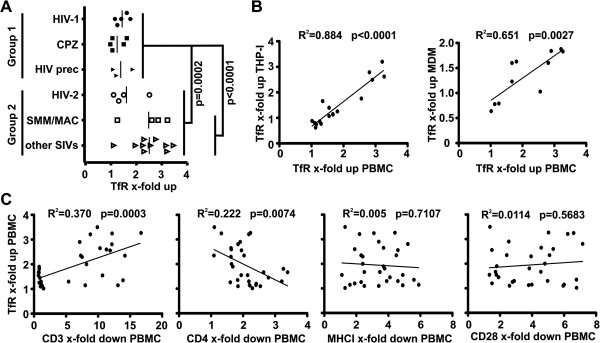
**Primate lentiviral Nef proteins differentially upregulate cell surface TfR. (A)** PBMC were infected with HIV-1 variants coexpressing 31 different primate lentiviral Nef proteins and GFP (compare Additional file 1: Table S1). TfR cell surface levels were measured by flow cytometry 48 hours post infection. Nef proteins were compared according to their phylogenetic relationship. Group 1 Nefs are derived from HIV-1 and its direct simian precursors whereas Group 2 Nefs represent all other lentiviruses including those which are non-pathogenic in their natural simian hosts. Each symbol represents the mean activity of a respective Nef protein in the function tested (please find the mean values ± SD in Additional file 1: Table S1). **(B)** THP-1 and monocyte derived macrophages (MDM) were infected with a subset of HIV-1 variants to analyse cell surface TfR modulation similar to the experiment described in **(A)**. The functional activity of the Nef proteins in upregulation of TfR in PBMC was correlated to the results from the THP-1 and MDM infection experiments. **(C)** PBMC infected with the 31 HIV-1 variants described in **(A)** were also analysed for Nef-mediated downregulation of cell surface CD3, CD4, MHCI and CD28. The results were correlated to the functional activity of the respective Nef proteins in upregulation of TfR.

To gain insights into the Nef-induced modulation of the TfR, we compared 31 *nef* alleles from HIV-1 M, O and N, SIVcpz, and the HIV-1 precursors SIVgsn/mus/mon (Group 1) as well as HIV-2, SIVsmm and divergent SIV species (Group 2, see also Additional file 1: Table S1). This collection faithfully represents a cross section of phylogenetically clustered and evolutionary related lentiviral *nef* alleles [10,14]. Isogenic HIV-1 NIG proviral constructs only differing in their respective *nef* ORF were used to generate virus stocks and infect PBMC, MDM and THP-1 cells (Figure 2). HIV-1 and related Nefs (Group 1) modulated TfR only marginally (1.39 ± 0.08; n = 13) in PBMCs (Figure 2A). In contrast, most other Nefs (Group 2) caused a marked increase in TfR expression at the cell surface (2.45 ± 0.18; n = 18). Of note, the only HIV-2 Nef that upregulated TfR is clone 60415 K (2.56 ±0.5, compare Additional file 1: Table S1) isolated from an apathogenic HIV-2 infection [17].

Given the critical role of macrophages in iron turnover *in vivo,* we next examined the effects of Nef in THP-1 cells and MDMs. We found that all *nef* alleles that modulated TfR surface levels in PBMCs were also active in THP-1 cells and MDMs (Figure 2B). Next, we tested in PBMCs whether Nef-induced upregulation of TfR surface levels correlates with other Nef functions (Figure 2C). Downmodulation of CD28 and MHCI by Nef did not correlate with TfR surface levels. In contrast, TfR upregulation exhibited a positive correlation with CD3 downmodulation and a negative one with CD4 downmodulation, although the R^2^ values were fairly low (R^2^ = 0.370 for CD3 and R^2^ = 0.222 for CD4; Figure 2C). These observations are in line with two previously reported observations: (i) CD3 is also modulated by Nef in a lineage-dependent manner [18] and (ii) TfR and CD4 are both internalized through a mechanism involving the clathrin adaptor protein 2 (AP2) [19,20]. However, in both cases we could also identify Nef proteins which were selectively defective in one of these functions or active in both. Thus, overlapping but distinct Nef regions seem to be involved in modulation of TfR, CD3 and CD4.

Our data reveal that Nef modulates TfR in a lineage dependent manner. HIV-1 and its simian precursors are inactive in this function whereas most other lentiviruses upregulate TfR.

### Tyrosine 28 in SIV Nef is required for TfR upregulation

**Figure 3:**
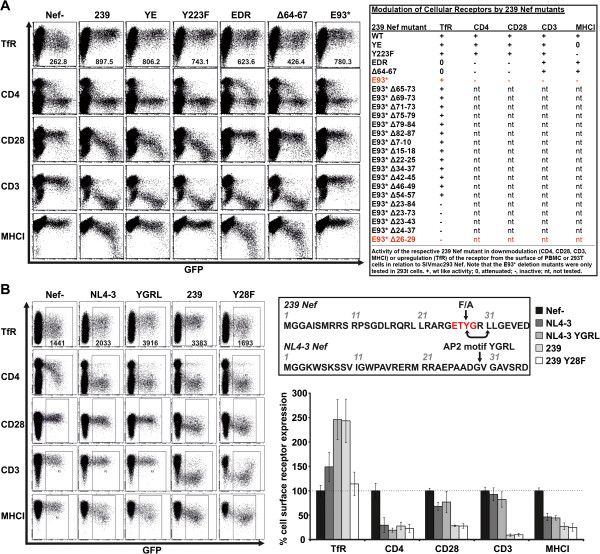
**An N-terminal AP2 binding motif is required and sufficient for upregulation of TfR by Nef. (A)** PBMC were infected with HIV-1 coexpressing GFP and various mutants of the SIVmac 239 Nef protein and assessed for TfR, CD4, CD28, CD3 and MHCI modulation by FACS 48 hours post infection. The table shows the qualitative activity of the Nef mutants in PBMC and the subsequent experiment of transfected 293 T cells with pCG-IRES-GFP expression plasmids of a truncated 239 Nef protein in combination with a variety of in-frame deletions. By this approach we could narrow down the critical domain for Nef-mediated TfR modulation to a four amino-acid deletion in the flexible N-terminal loop (Δ26-29). **(B)** PBMC were infected with HIV-1 NIG expressing the NL4-3 Nef or a variant in which we reconstituted the YGRL motif at position 28. In addition we infected PBMC with the HIV-1 NIG version of SIVmac 239 Nef and a mutant harbouring a change of Y28 to F. At 48 hours post infection modulation of TfR, CD4, CD28, CD3 and MHCI was assessed by flow cytometry. The graph shows mean values and SD of three independent infection experiments. For TfR modulation we have included the respective MFI of the infected GFP + cell population in the primary FACS plots.

To identify regions in Nef responsible for the increase in cell surface TfR, we tested a previously characterized [21] panel of SIVmac239 Nef mutants. PBMCs were infected with the HIV-1 NIG variants and analysed for modulation of several receptors (Figure 3A). The phenotype of the different 239 Nef variants for CD4, CD28, CD3 and MHCI modulation was as expected [21], although in this set of experiments Nef dependent modulation of CD4 is difficult to assess, since Vpu and Env also reduce CD4 expression in HIV-1 infected PBMC [22]. The YE and Y_223_ changes did not impair TfR upregulation whereas the EDR and Δ64-67 mutants were attenuated (Figure 3A). Of note, the truncated 239 Nef E_93_* selectively increased cell surface TfR. Thus, a region within the first 93 amino acids of SIVmac239 Nef is responsible for TfR upregulation.

To identify the Nef residues responsible for TfR upregulation we generated a panel of eighteen 239 Nef E_93_* mutants with serial deletions. Most deletions did not affect the capability of 239 Nef to increase TfR expression. However, a set of five mutants that lacked AA 26–29 were inactive (Figure 3A). This region comprises a tyrosine which is present in all Nef variants capable to increase TfR expression and is part of a canonical AP2μ binding site (Yxxϕ; ϕ is a bulky hydrophobic AA side chain; see Additional file 2: Figure S2 and [20,23]). Indeed, introduction of the “YGRL” motif in full-length NL4-3 Nef conferred the ability to increase cell surface expression of TfR in PBMC (Figure 3B). Conversely, mutation of 239 Nef Y_28_ to F (Figure 3B) or A (not shown) selectively disrupted the upregulation of TfR. In conclusion, a tyrosine based N-terminal AP2μ binding motif in Nef is critical for TfR upregulation.

### Strong Nef binding to AP2μ is associated with TfR upregulation

**Figure 4:**
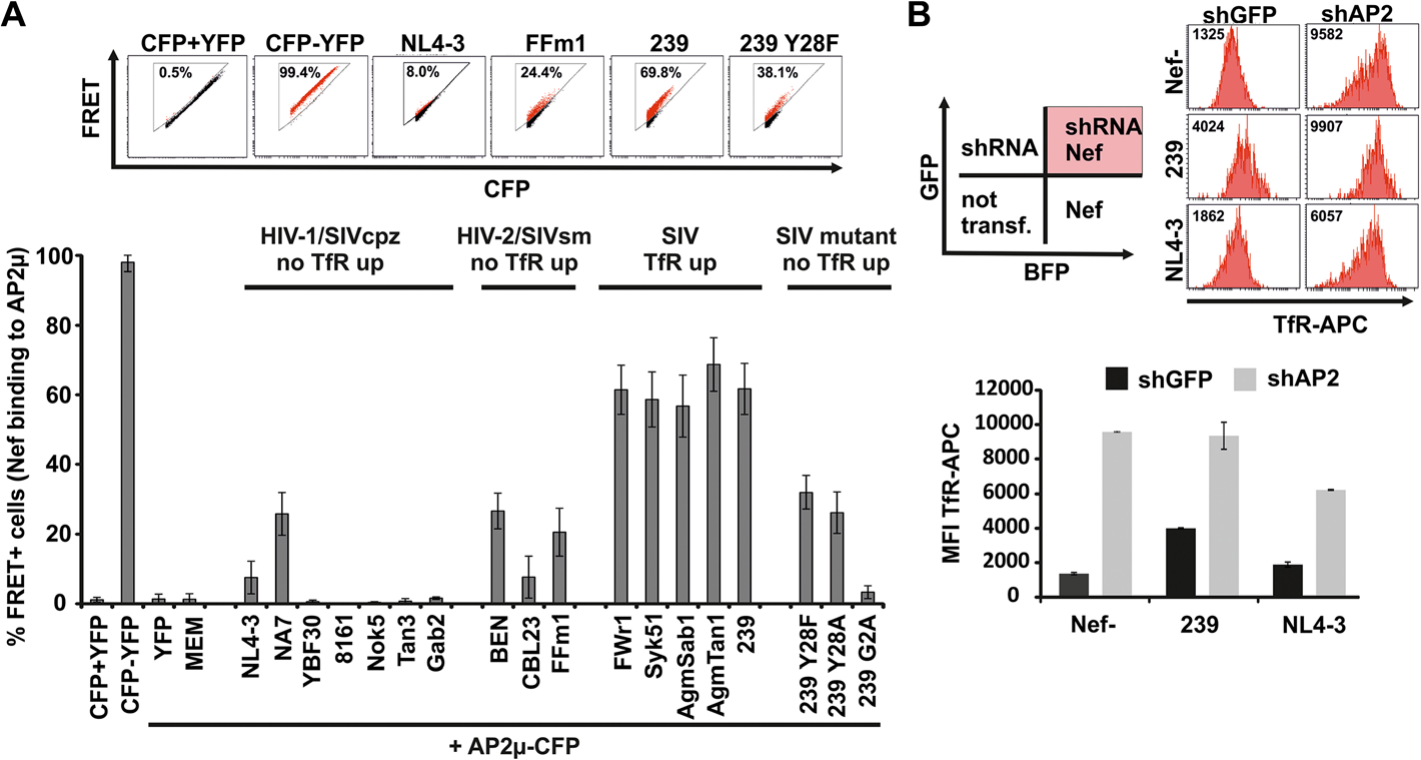
**Nef binding to AP2μ is associated with TfR upregulation. (A)** 293 T cells were cotransfected with different Nef-YFP fusion protein vectors and AP2μ-CFP. 24 hours later the amount of cells scoring FRET + was measured by flow cytometry as described before [24]. CFP + YFP is a negative control of 293 T cells which are cotransfected with CFP and YFP expressing plasmids. CFP-YFP is a positive control of 293 T cells that express a CFP-YFP fusion protein. Mean values and SD are calculated from four to nine independent transfections. **(B)** 293 T cells were transfected with lentiviral vectors coexpressing GFP and a shRNA against AP2μ or the GFP only expressing vector control. 72 hours later the cells were “supertransfected” with pCG-IRES-mTagBFP plasmids expressing different lentiviral Nef proteins. After additional 24 hours cells were stained with TfR-APC antibody and cell surface levels in shRNA/Nef expressing cells were assessed by flow cytometry. Mean values and SD from triplicate transfections of one representative experiment out of three is shown.

The canonical pathway of TfR internalization is by AP2 [20,23]. Hence, Nef-mediated AP2 sequestration might impede TfR uptake. To assess if lentiviral Nefs differentially interact with AP2μ we generated an AP2μ-eCFP fusion and Nef-eYFP fusion expression vectors and measured binding with a FACS-based FRET assay [24] (Figure 4A). Coexpression of HIV-1 and SIVcpz Nefs with AP2μ-eCFP did generally not result in FRET signals, implicating the absence of direct interaction with AP2μ (Figure 4A). In contrast, all HIV-2 and SIV Nefs showed FRET with AP2μ, although with considerable differences. Values higher than 40% of FRET + cells were exclusively observed for Nefs able to upregulate TfR whereas HIV-2 and SIV Nef variants unable to upregulate TfR or containing inactivating mutations exerted significant lower FRET (Figure 4A). Of note, the inactive HIV-2 Ben and SIVsm FFm1 as well as 239 Nef with the mutated YRGL motif still interacted with AP2μ, albeit mean FRET was lower than 35% in all measurements. This suggests (i) that other Nef residues contribute to AP2μ binding and (ii) that a certain threshold of AP2μ binding by Nef is required for increased TfR expression. In addition, differential Nef binding to other AP2 subunits (than μ) could also have an impact on TfR upregulation [25].

To further examine the role of AP2μ in Nef-mediated TfR upregulation, we performed AP2μ knock-down by shRNA. 293 T cells were transduced with self-inactivating lentiviral vectors expressing GFP as infection marker and a shRNA against AP2μ or a scrambled shRNA. Three days later, cells were transfected with pCG Nef expression vectors containing the fluorescence protein mtagBFP [26]. This strategy allows to specifically identify by FACS cells that simultaneously express the shRNA and Nef (Figure 4B). Only 239 Nef increased TfR in the presence of the control shRNA (black bars). Upon AP2μ knock-down, TfR accumulated at the cell surface irrespective of functional Nef expression. Altogether the data suggest that Nef sequesters AP2 resulting in reduced TfR internalization and surface accumulation.

### SIV Nef inhibits Transferrin internalization

**Figure 5:**
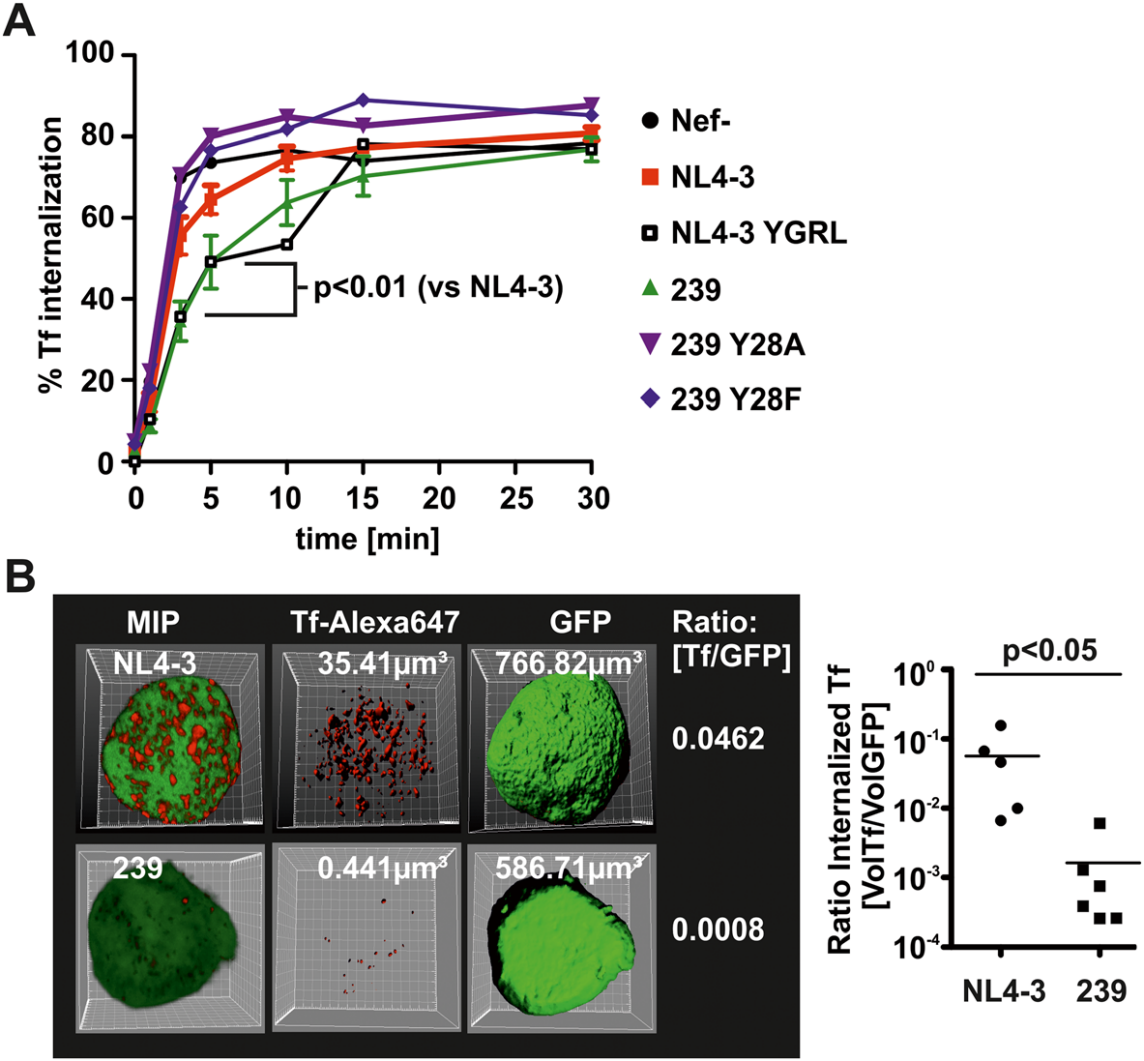
**SIV Nef inhibits Tf internalization. (A)** 293 T cells expressing the indicated Nef proteins were incubated with Alexa647-conjugated Transferrin for different periods of time. Then the cells were exposed to an acidic washing procedure to remove surface bound Tf and the amount of internalized Tf was determined by flow cytometry as described in detail in the methods section. Curves and SD were generated from three independent transfection procedures. **(B)** THP-1 monocytes were infected with HIV-1 NIG expressing NL4-3 Nef or the SIVmac 239 Nef. 48 hours post infection cells were incubated for 5 minutes with Alexa647-conjugated Transferrin. Then surface bound Transferrin was removed by acidic washing. Subsequently cells were fixed and z-stacks of GFP and Alexa674 fluorescence were recorded by confocal microscopy. 3D reconstruction shown as maximum intensity projections (MIP) and volume calculations were done using Bitplane Imaris version 6.4. The volume ratio of internalized Tf was calculated by dividing the Tf volume through the total volume of the cell, as assessed by GFP fluorescence.

Delayed TfR turnover should have direct impact on the internalization of Tf. Therefore, 293 T cells were transfected with pCG-Nef-IRES-GFP vectors and the amount of internalized Tf versus totally bound Tf was assessed in GFP/Nef expressing cells by FACS (Figure 5A). SIVmac239 Nef expression slowed down Tf internalization whereas disruption of the AP2μ binding motif (Y_28_F/A) abrogated this effect. In contrast, NL4-3 Nef had only minor effects on Tf internalization whereas introduction of the YGRL motif phenocopied SIVmac239 Nef.

To assess the consequence of delayed Tf internalization for the total amount of cellular Tf, we infected THP-1 cells with HIV-1 NIG and added Alexa647-Tf for 5 minutes. After removal of cell surface Alexa647-Tf by acid wash, z-stacks of GFP expressing/HIV-1 infected cells were acquired to reconstruct 3-dimensional images (Figure 5B). The total cellular volume was calculated using GFP fluorescence as a surrogate marker. Similarly, we calculated the volume of cell internal Tf from the Alexa647 fluorescence. Mean volume ratios [Tf/GFP] were 0.0572 (±0.0271 SEM; n = 5) for NL4-3 compared to 0.0015 (±0.0009 SEM; n = 6) for 239 Nef. This reflects a 38-fold reduction in the total volume of cell-associated Tf in SIVmac239 Nef expressing cells (Figure 5B) (p = 0.0043 Mann–Whitney test and p = 0.0497 Students T test). Thus, SIV Nef expression delays internalization of TfR and Tf which might reduce intracellular iron concentrations.

### SIV Nef lowers intracellular iron levels and attenuates viral replication in primary macrophages

The very small proportion of cellular free redox-active iron, called the labile iron pool (LIP), is subjected to rapid changes upon alterations in iron uptake or release [4]. Direct effects of Nef on the LIP were estimated using the green fluorescent dye calcein-acetoxymethylester (CA-AM) [4]. Calcein is quenched by free redox active iron. Thus, a decrease in Calcein fluorescence indicates an increase in the LIP. Unfortunately, due to the high Calcein background fluorescence and the small amount of labile iron, this assay exhibits a very low signal to noise ratio. Hence we expected small changes in fluorescence and the need for multiple biological replicates in order to achieve significant differences.

**Figure 6:**
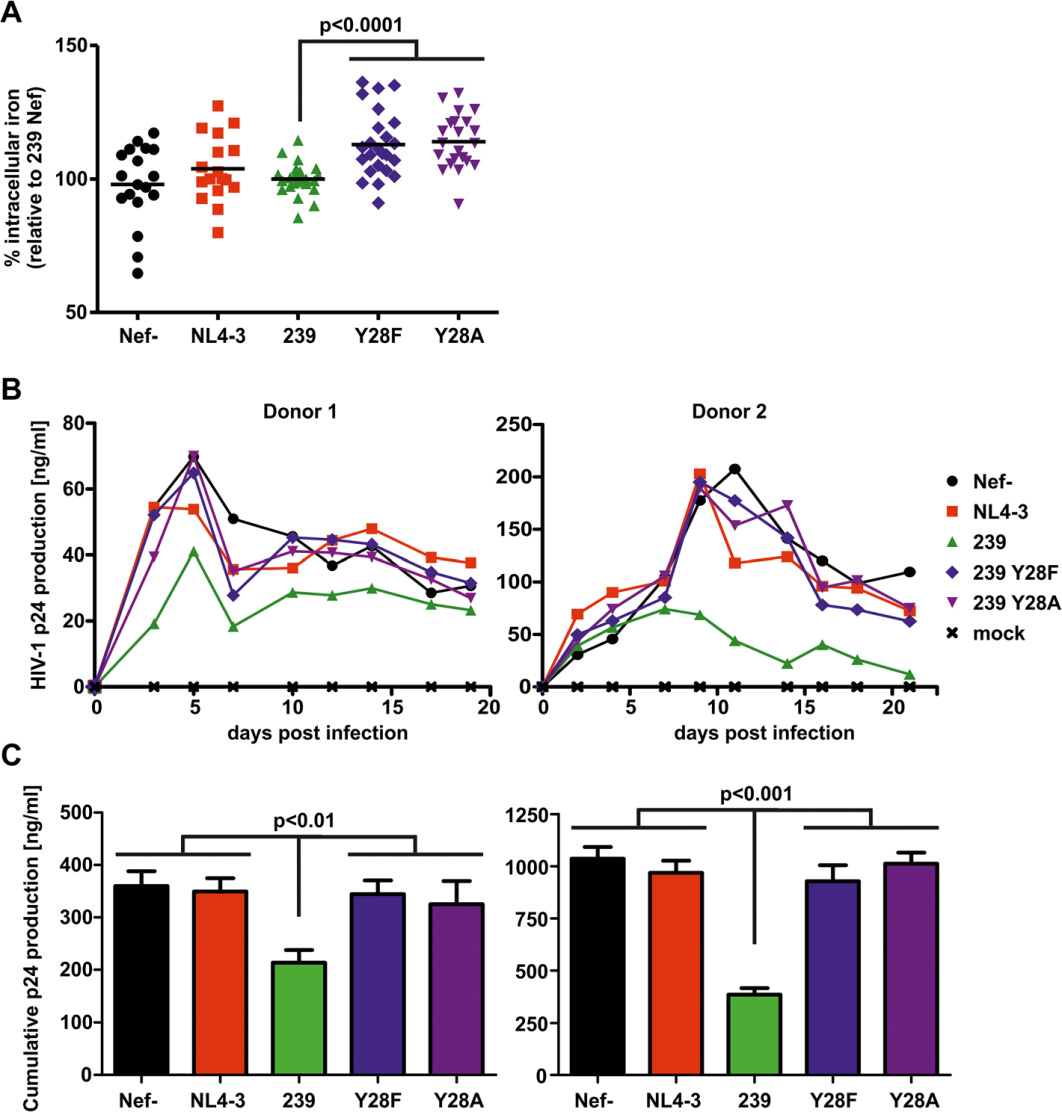
**SIV Nef decreases cellular iron levels and attenuates lentiviral replication in macrophages. (A)** THP-1 infected with HIV-1 expressing the indicated Nef proteins and eCFP via an IRES were stained with the green fluorescent dye CA-AM as described in the Methods Section. CA-AM is quenched upon the availability of chelatable iron within the cell. This decrease in fluorescence emission was assessed by FACS specifically in the HIV-1 infected and therefore eCFP positive population. Depicted is the increase in cellular chelatable iron relative to the 239 Nef expressing cells. Each symbol represents a single measurement from eight experiments with two to three independent virus stocks. **(B)** Primary macrophages from two donors were infected with 50 ng of normalized HIV-1 expressing the indicated Nef proteins. Aliquots of cell culture supernatants were taken in two to three day intervals and the amount of p24 production was measured by ELISA. The graphs show replication curves with mean values of four independent infections. **(C)** The cumulative p24 production is the absolute amount of released p24 during the culture period. Mean values and SEM for the two donors shown are calculated from the four independent infections. Similar results were obtained with macrophages from two other donors.

THP-1 cells were infected with HIV-NIG expressing eCFP instead of GFP. 48 hours later the LIP was analyzed post CA-AM staining in infected/eCFP expressing cells. We repeated the experiment eight times with two to three independent virus stocks and calculated the LIP relative to the Calcein fluorescence of SIVmac239 Nef positive cells (Figure 6A). THP-1 expressing no Nef or NL4-3 Nef showed a trend towards higher labile iron. However, Calcein fluorescence scattered strongly and differences were not significant. In contrast, SIVmac239 Nef expressing cells contained less labile iron than the Y_28_F/A mutants (Figure 6A) which do not block TfR internalization and Tf uptake. Despite the strong Calcein background fluorescence we measured an increase in mean values of 13.0% (239 Nef vs Y_28_F; n = 22) and 14.1% (239 Nef vs Y_28_F; n = 22) which is highly significant (p < 0.0001). Hence, Nef proteins that inhibit Tf uptake lower the amount of redox active iron within the cell.

Macrophages are important HIV-1 target cells *in vivo* and play a key role in cellular iron metabolism [1,15]. Since iron is an essential cofactor for multiple steps in the viral replication cycle [2], we speculated that differences in cellular iron uptake due to Nef expression might influence the capacity of HIV-1 to replicate in primary macrophages. MDMs were infected with isogenic R5-tropic HIV-1 just differing in the *nef* coding sequence and virus production was monitored over twenty days (Figure 6B and C). As expected from previous results [27], NL4-3 Nef expression had no influence on HIV-1 spread and propagation in macrophages. In contrast, 239 Nef suppressed HIV-1 replication in macrophages and this effect was dependent on Y_28_ (Figure 6B and C). Therefore, 239 Nef might attenuate lentiviral replication in macrophages by lowering the intracellular iron pool.

## Discussion

Our study suggests that manipulation of cellular iron uptake is a strategy of primate lentiviruses to regulate replication in myeloid cells. Moreover, the Transferrin receptor pathway is not inhibited by HIV-1 and its precursors but by most other SIV Nefs.

Previous work suggested HIV-1 Nef mediated downregulation of HfE [9] and TfR [28]. We show that HIV-1 Nefs have little if any effect on cell surface TfR while most SIV Nef proteins caused an up to three fold upregulation (Figure 2 and Additional file 1: Table S1). At first, these discrepancies seem surprising. However, most previous results were performed in HeLa cells overexpressing Nef and quantification was mainly performed by confocal microscopy [28]. In contrast, the majority of our experiments were performed using flow cytometry and conducted in HIV-1 infected PBMCs and macrophages. Furthermore, our finding that HIV-1 Nef does only marginally manipulate TfR is in line with others [18,29,30]. For detection of HfE, Drakesmith and colleagues used HfE overexpressing HeLa cells and HIV-1 SF2 Nef [9]. We mimicked this experiment and included HCMV US2 as positive control (Figure 1B) [16]. HfE was modulated by SF2 Nef (~40%), but not by any other Nef protein. In contrast, HCMV US2 degraded more than 90% of HfE. Thus, downregulation of HfE is a less pronounced and non-conserved Nef function.

Conversely, plasma membrane levels of TfR increased up to 3.5 fold post SIV Nef expression in infected PBMCs and were on average elevated 2.5 fold which is in the range of the well characterized Nef functions CD4 and MHC-I downregulation (Figure 2, Additional file 1: Table S1; also reviewed here [10]). Mechanistically, several lines of evidence suggest that SIV Nef upregulates TfR by competition for AP2: (i) We could identify a conserved Yxxϕ sequence which is a canonical AP2μ binding motif [20,23] and present in the N-terminus of all Nefs upregulating TfR (Additional file 2: Figure S2). (ii) Mutation of Y_28_ selectively disrupts Nef-mediated TfR increase (Figure 3B). (iii) Insertion of the Yxxϕ in HIV-1 Nef confers upregulation of TfR (Figure 3B). (iv) The capacity to increase cell surface TfR is associated with strong Nef binding to AP2μ (Figure 4A) and (v) knock-down of AP2 by shRNA increases TfR irrespective of Nef expression (Figure 4B). Thus, the data indicates that SIV Nef sequesters AP2 from TfR and “upregulation” of TfR is an effect of the decelerated internalization of the receptor and decreased cellular Tf uptake (Figure 5A). Importantly, steady state expression of TfR at the PM might strongly underestimate the magnitude of repressed cellular Tf uptake reflected by our 3D reconstructions (Figure 5B). Thus, lentiviral Nef proteins inhibiting TfR internalization will strongly suppress cellular Tf and iron uptake.

What are the consequences of dysregulated iron homeostasis in lentiviral infections? Iron is important for an effective immune response [1-3]. However there is some controversy because increased iron levels correlate with severe HIV-1 progression [31-36], whereas progressive HIV-1 infection is also associated with anemia and therefore iron depletion [37,38]. Furthermore, HIV-1 patients with elevated cellular iron stores showed strongly reduced survival probabilities [6-8,39,40].

In this context it is noteworthy that generalized unspecific immune activation is a hallmark of progressive HIV-1 infection [13]. Thus, infected immune cells are in a state of hyperactivation and cellular iron is increased upon inflammation which is beneficial for retroviral replication [5]. Multiple steps in the lentiviral life cycle are iron dependent. Among them provision of nucleotides, NF-κB activation [41], Tat dependent transcription [42], Rev mediated mRNA export [43] and final steps of assembly and release [44] (reviewed in [2,8]). Conceivably, iron chelation *in vitro* inhibits HIV-1 infection and replication [41-43,45]. Considering the high iron need of HIV-1 in conjunction with the appearance of opportunistic infections that also consume iron for growth, anemia and iron depletion in progressive HIV-1 infection is not surprising [46]. Whether dietary iron supplementation at that stage might be helpful for the host or rather deleterious is a question of high relevance that is currently not yet answered [47].

Setting up iron limiting experimental conditions *ex vivo* is difficult. Nevertheless we could demonstrate the direct reduction of labile iron by HIV-1 expressing SIVmac 239 Nef. This phenotype correlated with an attenuated course of viral replication in macrophages (Figure 6). In contrast, HIV-1 replication and CD4+ T cell depletion in *ex vivo* cultures of lymphoid tissue were not affected by mutation of Y28 in 239 Nef (see Additional file 3: Figure S3). Hence, regulation of cellular iron uptake could represent a strategy of lentiviruses to control virus growth in certain infected cells or tissue, i.e. macrophages. TfR internalization was only inhibited by Nefs from lentiviruses that are highly divergent from HIV-1 including those which are most likely apathogenic in their natural simian hosts. These SIVs preferentially use CCR5 for cell entry and since their genomes express Vpx, the cellular tropism of the virus is greatly expanded allowing efficient infection of myeloid cells [48]. Under these conditions it might have been necessary to maintain a function that limits excessive replication in myeloid/macrophage cells. Of note, robust SIV replication in macrophages was observed in the SIV macaque model and has also been associated with progression of simian AIDS [49]. In this context, the SIV Nef mutants which are selectively defective for block of TfR internalization identified herein are valuable tools to clarify the impact of altered cellular iron on pathogenicity and viral loads in a model of natural SIV-infected monkeys.

Reduction of cellular iron was only observed for SIV Nef. In general, HIV-1 and related Nefs had only marginal effects on TfR and HfE cell surface levels. Therefore HIV-1 does either not manipulate cellular iron or has evolved differential mechanisms to dysregulate iron homeostasis. Given the importance of iron for lentiviral growth, it will be of high interest to investigate in future studies possible alternative mechanisms by which HIV-1 could alter cell associated iron.

## Conclusions

Herein we establish inhibition of TfR internalization as a novel function that is exerted by most SIV Nef proteins in primary T cells and macrophages. We identify an YXXϕ AP-2 binding motif in the N-terminus of Nef that is sufficient and necessary to confer this function. Nef competes with AP-2 for TfR binding resulting in reduced internalization of Transferrin and therefore iron delivery into the cell. In myeloid cells we could demonstrate the direct reduction of iron uptake leading to attenuated lentiviral replication in this cell type. We postulate that reduction of cellular iron uptake by SIV Nef is a function that evolved to regulate viral replication in macrophages and this could have an impact on lentiviral pathogenicity.

## Methods

### Proviral constructs and plasmids

HIV-1 pBR-NL4-3 IRES-eGFP proviral constructs expressing different lentiviral Nefs have been described previously (NL4-3 NIG) [14]. R5-tropic derivatives of these constructs were generated by subcloning of the *nef* ORF via *HpaI* and *MluI* into pBR-NL4-3 V3 92th014.12-IRES-eGFP [50]. Similarly, HIV-1 NIG variants expressing different SIVmac 239 Nef mutants were generated by PCR amplifications from pCG-vector templates that were published previously [21]. The SIVmac239 Nef Y_28_A/F mutants were generated by splice overlap extension PCR and the YGRL sequence was introduced at position 28 in NL4-3 Nef by primer mutagenesis. CMV-driven pCG plasmids coexpressing Nef and GFP via an IRES have been described before [51]. pCG-SF2-*nef*-IRES GFP and the pCG-HCMV-*US2*-IRES GFP were generated by amplification of the specific reading frame with primers introducing 5′ *XbaI* and 3′ *MluI* restriction sites and subsequent standard restriction and ligation procedures. Truncated pCG-SIVmac 239 Nef variants with different in frame deletions were generated by PCR amplification and ligation of published deletion mutants [19] with primers introducing a 5′ *XbaI* site and a premature stop codon at aa position 93 followed by a 3′ *MluI* site. Fusion protein vectors peCFP and peYFP, peCFP-eYFP, NL4-3 Nef-YFP and SIVmac 239 Nef-YFP have been described before [24]. Plasmids expressing AP2μ and HfE with a C-terminal CFP-tag were constructed by PCR-amplification of Ap2μ and HfE from a HeLa cDNA library. Lentiviral Nef-YFP fusion proteins were amplified using the HIV-1 NIG proviral vectors as templates. The according ligation procedure has been described [24]. All PCR derived inserts were sequenced to confirm sequence identity.

### Cell culture, transfection and HIV-1 infection

293 T cells were maintained in DMEM (Gibco) and THP-1 cells in RPMI (Gibco) with standard supplements. Primary human monocyte derived macrophages (MDM) and primary blood derived mononuclear cells (PBMC) were isolated and cultured as described [14,50]. HIV-1 virus stocks were generated by calcium phosphate transfection of 293 T cells [14]. For infection experiments HIV-1 stocks were quantified by p24-ELISA [27]. To assess HIV-1 replication in macrophages 25.000 cells were seeded in 48 well plates in 1% serum conditions and infected with 50 ng p24. Six hours later cells were washed and new media was added. Aliquots were taken in two to three days intervals and virus production was quantified by p24 ELISA. For the flow cytometric measurements of cell surface receptor modulation 2*10^5 macrophages or 1*10^6 PBMC were seeded in 35 mm Greiner dishes or six well plates and infected with 200 ng p24. Macrophages were harvested five days later by 10 mM EDTA treatment. PBMCs were usually analysed 48 hours post infection. HIV-1 infection experiments of human lymphoid tissue (HLT) was done as already described [27].

### Flow cytometry

Antibody staining was done on ice. Cells were washed once with PBS/1% FCS and afterwards incubated for 30 minutes in a total volume of 100 μl with the respective recommended amount of antibody. Following antibodies were used in our study: anti-CD71 (BDPharmingen; M-A712; APC), anti-CD28 (BDPharmingen; L293; PE), anti-HFE (Abnova; polyclonal), anti-CD3 (Caltag; UCHT1; APC), anti-CD4 antidody (Caltag; RPA-T4; APC), anti-HLA-ABC (Dako; W6/32; PE). Post staining cells were washed twice and fixed with 2% PFA/PBS. At least 2.000 infected cells were measured with a FACS Canto II (BDBioscience). Fold modulation of cell surface receptor modulation was calculated as before [14,51]. Flow cytometric measurement of FRET and the according gating strategy were performed as already described [24]. Cellular iron content was measured by the use of the fluorescent dye CA-AM which is quenched by free cellular iron [4].

### Internalization assay

Tf internalization assays were performed essentially as previously described [18]. In brief, cells were harvested and incubated with Tf-Alexa647 for 30 min on ice, washed, and shifted to 37°C for various periods of time in culture medium supplemented with 20 mM HEPES. The medium was removed by washing, and half the samples were washed in 25 mM glycine-HCl–125 mM NaCl (pH 2.8) and rapidly neutralized with 25 mM Tris (pH 10). Samples were then washed and analyzed by flow cytometry. Mean fluorescence intensities (MFI) of GFP-positive cells in FL4 were determined. The ratio of the intracellular MFI (acid wash resistant) to the total MFI at each time point was plotted as a function of time.

### Image acquisition, analysis and software

Infected THP-1 cells were incubated with Alexa647 labeled Tf for 5 minutes. Then we exposed the cells to the acidic washing procedure as described above and finally fixed them with 2% PFA. THP-1 cells were mounted on objective slides with Mowiol and z-stacks of infected GFP expressing cells were acquired with a Zeiss LSM510 Meta. 3D reconstruction of z-stacks was done with Bitplane Imaris V6.4.2. We also used this software to calculate the volume of the cells by GFP expression and the volume of internalized Tf by Alexa647 fluorescence. In general, images were never modified apart from enhancing contrast and/or brightness. Statistical analyses were performed using the GraphPad Prism V5 software package. Statistical tests used were the unpaired two-tailed T test and the Mann–Whitney test and regression analyses with p-value calculations.

## Competing interests

The authors declare that they have no competing interests.

## Authors’ contributions

Designed research: HK, KH, AH, PB, MS; Performed research: HK, KH, MVG, FXG, MW, SG, AH, MS; Contributed reagents: HK, MWK, FK, PB, MS; Analyzed data: HK, PB, MS; Wrote the manuscript: MS. All authors read and approved the final manuscript.

## Supplementary Material

Additional file 1: Table S1 Modulation of TfR and previously described PBMC surface receptors by lentiviral Nef proteins. Click here for file

Additional file 2: Figure S2 Alignment of the N-terminus of Nef variants analyzed. The first 70 aminoacids of all lentiviral Nefs analyzed in this study were aligned. The tyrosine of the putative AP2-binding motif Yxxϕ is marked in red. All Nef variants inactive in TfR upregulation are grouped by the red square whereas Nef proteins inhibiting TfR uptake are surrounded by the green square. Click here for file

Additional file 3: Figure S3 SIV 239 Nef with or without mutated Y28 motif does not affect HIV-1 replication and CD4+ T cell depletion in *ex vivo* infected human lymphoid tissue (HLT). Cumulative p24 production over 13 days (left) and CD4+ T cell depletion at the end of the culture period (right) in tissues of five donors infected with the indicated R5-tropic HIV-1 NL4-3 variants. Shown are mean values +/- SEM. Click here for file
