# Supplementary material for: Lentiviral Nef suppresses iron uptake in a strain specific manner through inhibition of Transferrin endocytosis
Source: Retrovirology. 2014 Jan 2;11:1. doi: 10.1186/1742-4690-11-1 (PMC3892060; doi:10.1186/1742-4690-11-1)
Supplement: Additional file 1: Table S1 — Modulation of TfR and previously described PBMC surface receptors by lentiviral Nef proteins. [file 1742-4690-11-1-S1.pdf]

Supplemental Table S1. Modulation of TfR and previously described PBMC surface receptors by lentiviral Nef proteins.

Lineage	Clone	Species/subspecies	Modulation (n-fold, ±SD) of: TfR[(1)]	CD4[(2)]	MHCI[(2)]	CD3[(2)]	CD28[(2)]	Group[(3)]	Ref
HIV-1/M	NL4-3	Human (*Homo sapiens*)	1.67 ±0.3	2.6	3.4	0.8	1.7	1	14
HIV-1/M	NA7	Human (*Homo sapiens*)	1.81 ±0.4	3.3	3.7	0.8	3.8	1	14
HIV-1/O	MVP8161	Human (*Homo sapiens*)	1.45 ±0.3	2.8	4.1	0.9	1.9	1	14
HIV-1/O	MVP13127	Human (*Homo sapiens*)	1.30 ±0.3	3.2	4.6	1.1	2.9	1	14
HIV-1/N	YBF30	Human (*Homo sapiens*)	1.20 ±0.2	2.4	1.9	1.1	1.5	1	14
SIVcpz*Ptt*	GAB2	Central chimpanzee (*Pan t. troglodytes*)	1.67 ±0.2	3.4	2.5	0.7	4.5	1	14
SIVcpz*Ptt*	Cam3k1	Central chimpanzee (*Pan t. troglodytes*)	1.55 ±0.3	2.1	1.1	0.7	1.3	1	14
SIVcpz*Pts*	TAN1	Eastern chimpanzee (*Pan t. schweinfurthii)*	1.02 ±0.1	2.3	1.3	1.5	6.8	1	14
SIVcpz*Pts*	TAN3	Eastern chimpanzee (*Pan t. schweinfurthii)*	1.10 ±0.4	1.8	2.5	1.0	2.6	1	14
SIVcpz*Pts*	Nok5	Eastern chimpanzee (*Pan t. schweinfurthii)*	1.10 ±0.4	2.5	3.4	1.1	5.9	1	14
SIVgsn	CM166	Greater spot-nosed monkey (*C. nictitans*)	1.13 ±0.1	2.2	4.9	1.3	3.8	1	14
SIVmus	CMS1085	Mustached monkey (*Cercopithecus cephus*)	1.90 ±0.4	1.7	3.7	0.8	1.8	1	14
SIVmon	CML1	Mona monkey (*Cercopithecus mona*)	1.26 ±0.2	1.6	2.3	0.8	2.8	1	14
HIV-2	BEN	Human (*Homo sapiens*)	1.35 ±0.2	1.8	5.6	13.9	6.8	2	14
HIV-2	CBL-23	Human (*Homo sapiens*)	1.15 ±0.5	2.2	5.9	13.1	4.0	2	14
HIV-2	60415K	Human (*Homo sapiens*)	2.56 ±0.5	2.0	4.6	11.9	1.5	2	14
HIV-2	310319	Human (*Homo sapiens*)	1.55 ±0.1	1.9	4.9	10.7	1.8	2	14
SIVsmm	FFm1	Sooty mangabey (*Cercocebus atys*)	1.30 ±0.1	2.2	3.9	5.4	5.0	2	14
SIVsmm	FYr1	Sooty mangabey (*Cercocebus atys*)	2.65 ±0.1	1.6	3.7	10.3	4.6	2	14
SIVsmm	FWr1	Sooty mangabey (*Cercocebus atys*)	2.90 ±0.3	1.9	3.4	7.1	2.6	2	14
SIVmac	239	Rhesus macaque (*Maccaca mulatta*)	3.27 ±0.4	2.2	4.4	16.7	5.0	2	14
SIVrcm	GB1	Red-capped mangabey (*Cercocebus torquatus*)	3.50 ±0.1	1.1	1.3	9.9	1.4	2	14
SIVdeb	CM5	De Brazza monkey (*Cercopithecus neglectus*)	1.15 ±0.2	2.4	1.6	8.0	6.0	2	14
SIVdeb	CM40	De Brazza monkey (*Cercopithecus neglectus*)	2.00 ±0.1	1.6	2.4	8.2	4.8	2	14
SIVsyk	KE44	Sykes` monkey (*Cercopithecus albogularis*)	2.30 ±0.1	1.6	2.4	7.5	3.9	2	14
SIVsyk	KE51	Sykes` monkey (*Cercopithecus albogularis*)	3.25 ±0.1	1.3	3.1	12.2	6.6	2	14
SIVblu	KE31	Blue monkey (*Cercopithecus mitis*)	2.81 ±0.2	1.8	5.2	12.1	7.2	2	14
SIVsun	sol-36	Sun-tailed monkey (*Cercopithecus solatus)*	2.35 ±0.3	2.0	2.3	7.3	2.4	2	14
SIVtan	1	Tantalus monkey (*Chlorocebus tantalus*)	3.21 ±0.5	2.2	2.2	8.8	6.2	2	14
SIVtan	B87-18	Tantalus monkey (*Chlorocebus tantalus*)	2.60 ±0.2	1.6	1.9	11.3	5.3	2	14
SIVsab	1	Green monkey (*Chlorocebus sabaeus*)	2.35 ±0.1	1.1	4.5	14.2	5.1	2	14

The Nef-mediated *n*-fold modulation of TfR, CD4, MHCI, CD3 and CD28 was calculated by dividing the mean fluorescence intensity (MFI) of PBMCs infected with a *nef*-deficient HIV-1 expressing GFP by the corresponding MFI obtained for cells infected with HIV-1 constructs coexpressing Nef and GFP. For TfR results are shown in mean values ±SD from three to five independent experiments. [(1)]TfR is upregulated while [(2)]CD4, MHCI, CD3 and CD28 are downmodulated. [(3)]Phylogenetical clustering of lentiviral Nef proteins in HIV-1/SIVcpz and SIVprecursors (Group 1) versus HIV-2/SIVsmm/SIVmac and other SIVs (Group 2) according to Ref 14.
