# Supplementary material for: Lentiviral Nef suppresses iron uptake in a strain specific manner through inhibition of Transferrin endocytosis
Source: Retrovirology. 2014 Jan 2;11:1. doi: 10.1186/1742-4690-11-1 (PMC3892060; doi:10.1186/1742-4690-11-1)
Supplement: Additional file 2: Figure S2 — Alignment of the N-terminus of Nef variants analyzed. The first 70 aminoacids of all lentiviral Nefs analyzed in this study were aligned. The tyrosine of the putative AP2-binding motif Yxxϕ is marked in red. All Nef variants inactive in TfR upregulation are grouped by the red square whereas Nef proteins inhibiting TfR uptake are surrounded by the green square. [file 1742-4690-11-1-S2.pdf]

```
            1          11         21         31         41         51         61
    NL43  MGGKWSKSSV IGWPAVRERM RRA------- -EPAAD---G VGAVSRDLEK HGAITSSNTA ANNAAC----
     Na7  .......R.A G..S...K.. EQ.------- -.....---. .......... Y......... T...D.----
    Gab2  ..N......I V...Q....L ..T------- -QE..E---. ..E..Q..AR R.....RH.P QT.QTL----
    Cam3  ..NR.....L V...D....L ..T------- -Q...P---. ..P..Q...A ...V.TRH.P Q..QTL----
   YBF30  ..KI.....L ....EI.... ..QTQ----- -...VEPAV. A..A.Q..AN R....IR..R D..ESI----
    8161  ..NVLG.GKF E..SR..... .KTT------ -PEPEPCAP. ..QI.QE.AA R.G.PN.Y.P Q....L----
   13127  ..NVLT..QF P..AT...K. ..TSRRTSRT PPEPEPCAP. ..QI..E.AA R.G.P..Y.P Q....L----
    Tan1  ..NIFGR--W P.ARKAI.DL HNT------- --SSEP---- ..QA.Q..QN K.GL.TNTLG TSADVL----
    Tan3  ..NIFG.--W P.AQRAI.EL HKS------- --SHEP---- ..QA.K..QN R.GL.TNTIG TPEDVL----
    Nok5  ..SA...IKW V.ARQAIRKI HET------- --NPD.---- I.PCGKE.AS R..L...TIG TEKDVI----
     GSN  ..S.N..QQQ QESSTALLSS .GTGQRPYFT LVDEYGENFW LSPDAS.KGR RYYL.E--EP KPKRG-----
     Mon  ..S.S..QQS PLSSTPLLGS QSSGRMRYFM L.DDYGEQSW LSPDAS.R.R KYSL.EGRNG KQRRQ-----
     Mus  ..S.S..QPA QQSLISSPPS PGTGRKQYFK LVEEYGENSW LSPDASGRGR RYSL.E---G .SKRP-----
     Ben  ..ASG..KLS KHSRGL...L L..RGDGYGK QRD.SGGEYS QFQEESGR.Q NSPSCEGQQY QQGEYMNTPW
   CBL23  ..ASG..ERS RPSQGL...L L..RGETCGG HCSGSGGEYL QSQEESGR.Q RSPSYEGQRY QQGDFMNTPW
  310319  ..SAG..KQS KQQQGL...L L..RRGHYGE LSGEQREGSL RSPGES.KGL NSPSCEGQKT LGAEGGG---
    FFm1  ...VT..KQR KRGGNL...L LQ.RGETCGK LWDGSEGEYL QFQDASGRGL SSLSCEPQRY CEGQFMNTPW
     CM5  ....N..TPS F.QGKKSGSG SKGLLRWRYK DLSGEAETFS PFLQES.K.Q SCYS.EEPLY .DPHRE----
```

```
   60414  ..ASG..KRS RQPRGLQ..L L..RAGACGG YWNESGGQYS RSQEGSGR.Q RSPSCEGQRY QQGDFMNTPW
    FYr1  ...VT..KQS RPQREL..KL LQ.RGETYGR LWEGLEEGYS QSRGGLGRDW SLRSCEGQKY SEGQFMNTPW
    FWr1  ...VT..KQS KRRQGL...L LQ.RGETYGR LWEGLEDGYS RSRGELGKGW NLPSCEGQGY SEEQFMNTPW
     239  ...AI.MRRS RPSGDL.Q.L L..RGETYGR LLGEVEDGYS QSPGGL.KGL SSLSCEGQKY NQGQYMNTPW
     RCM  ....S..N.A A.LLRW.FK. LTTPGEGYVR WHETLLDGQP WC.EGSGRAS RDFVIRGGIT .ETQ.S----
 Agmtan1  ...SN..REQ Q.LLRLWRAL .K.PVVRYGM LADPLIGQSS NIQEEC.KNW N.GS.R--RG KSTPEG----
Agmtan18  ...SN..KEQ E.LLKMWRL. .K.PVVRYDM LADPLIGTSS SIQEEC.KNW S.GL.K-GKG KMTPEG----
  Agmsab  ....S..QQQ RHSLWLWSKL .Q.PVIQYDM LADPLLGQSS HIQEECAKSL RDGLIRQGDS SRTEEG----
    CM40  ....N..IPS R.QVGSFGSG S.GLLRWRYR DLSEQEEQFS ECLLES.R.Q SSSS.E--FW GSPRRE----
   Syk44  ..SVK.RMQQ SA.EDRL.TG WWKRRGKYTP FPD.LLRASL PSRGGF.KAW RSTL.EPIDP HGPDRD----
   Blu31  ..STS..NQQ CRSESPYGTR WWRRAKQYTP LPDELLKPSR PSHGGF.KAW RSTL.EPVDP HGPDRD----
   Syk51  ..SQS..KQP SR.DEKWRTR WWPFGKPYSP MPDELLRMSQ PYHEDF.RGW RSTL.EPI-- LDPKRD----
   Sun36  ..NAFG.P.E V..VRTLFKL .AGSGTRAEP AGR.YHRIR. ETEPLRSNPD G.LEELEE.E VGFPVRPQRP
```

Supplemental Figure S2: Alignment of the N-terminus of Nef variants analyzed. The first 70 aminoacids of all lentiviral Nefs analyzed in this study were aligned. The tyrosine of the putative AP2-binding motif Yxxϕ is marked in red. All Nef variants inactive in TfR upregulation are grouped by the red square whereas Nef proteins inhibiting TfR uptake are surrounded by the green square.
