# Supplementary material for: Lentiviral Nef suppresses iron uptake in a strain specific manner through inhibition of Transferrin endocytosis
Source: Retrovirology. 2014 Jan 2;11:1. doi: 10.1186/1742-4690-11-1 (PMC3892060; doi:10.1186/1742-4690-11-1)
Supplement: Additional file 3: Figure S3 — SIV 239 Nef with or without mutated Y28 motif does not affect HIV-1 replication and CD4+ T cell depletion in ex vivo infected human lymphoid tissue (HLT). Cumulative p24 production over 13 days (left) and CD4+ T cell depletion at the end of the culture period (right) in tissues of five donors infected with the indicated R5-tropic HIV-1 NL4-3 variants. Shown are mean values +/- SEM. [file 1742-4690-11-1-S3.pdf]

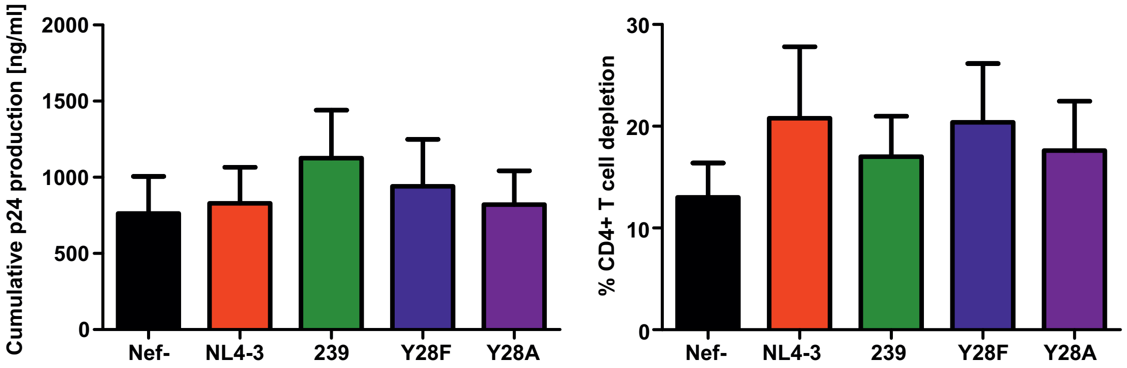

Supplemental Figure S3: SIV 239 Nef with or without mutated Y28 motif does not affect HIV-1 replication and CD4+ T cell depletion in *ex vivo* infected human lymphoid tissue (HLT). Cumulative p24 production over 13 days (left) and CD4+ T cell depletion at the end of the culture period (right) in tissues of five donors infected with the indicated R5-tropic HIV-1 NL4-3 variants. Shown are mean values +/- SEM.
